# High peak inspiratory pressure may be associated with intraoperative coughing during neurosurgery under general anesthesia without neuromuscular blockade: a retrospective study

**DOI:** 10.1186/s12871-023-02080-6

**Published:** 2023-04-14

**Authors:** Hyongmin Oh, Jin Young Sohn, Seoyoung Ma, Seungeun Choi, Yoon Jung Kim, Hyung-Chul Lee, Chang-Hyun Lee, Chi Heon Kim, Chun Kee Chung, Hee-Pyoung Park

**Affiliations:** 1 Department of Anesthesiology and Pain Medicine, Seoul National University Hospital, Seoul National University College of Medicine, 101, Daehak-ro, Jongno-gu, Seoul, 03080 Republic of Korea; 2 Department of Neurosurgery, Seoul National University Hospital, Seoul National University College of Medicine, 101, Daehak-ro, Jongno-gu, Seoul, 03080 Republic of Korea; 3 Department of Brain and Cognitive Sciences, Seoul National University, 1, Gwanak-ro, Gwanak-gu, Seoul, 08826 Republic of Korea

**Keywords:** Intraoperative coughing, Neurosurgery, Peak inspiratory pressure

## Abstract

**Background:**

The endotracheal cuff pressure depends on the airway pressure during positive-pressure ventilation. A high endotracheal cuff pressure may be related to intraoperative coughing, which can be detrimental during neurosurgery. We investigated the incidence of intraoperative coughing and its association with peak inspiratory pressure (PIP) during neurosurgery under general anesthesia without neuromuscular blockade.

**Methods:**

This retrospective study divided 1656 neurosurgical patients who underwent total intravenous anesthesia without additional neuromuscular blockade after tracheal intubation into high (PIP > 21.6 cmH_2_O, n = 318) and low (PIP ≤ 21.6 cmH_2_O, n = 1338) PIP groups. After propensity score matching, 206 patients were selected in each group. Demographic, preoperative, surgical, and anesthetic data were collected retrospectively from electronic medical records and continuous ventilator, infusion pump, and bispectral index data from a data registry.

**Results:**

Intraoperative coughing occurred in 30 (1.8%) patients, including 9 (0.5%) during the main surgical procedure. Intraoperative coughing was more frequent in the high PIP group than in the low PIP group before (14/318 [4.4%] vs. 16/1338 [1.2%], P < 0.001) and after (13/206 [6.3%] vs. 1/206 [0.5%], P = 0.003) propensity score matching. In multivariable logistic regression analysis after propensity score matching, a high PIP (odds ratio [95% confidence interval] 14.22 [1.81-111.73], P = 0.012), tidal volume divided by predicted body weight (mL/kg, 1.36 [1.09–1.69], P = 0.006), and surgical duration (min, 1.01 [1.00–1.01], P = 0.025) predicted intraoperative coughing.

**Conclusion:**

The incidence of intraoperative coughing was 1.8% in neurosurgical patients undergoing general anesthesia without neuromuscular blockade and might be associated with a high PIP.

**Supplementary Information:**

The online version contains supplementary material available at 10.1186/s12871-023-02080-6.

## Introduction

An endotracheal tube is commonly inserted and maintained for mechanical ventilation during surgery under general anesthesia. However, it can induce some discomfort secondary to tracheal mucosa irritation, resulting in coughing or bucking. During neurosurgery, the risk for intraoperative coughing is relatively high, because the use of neuromuscular blocking agents (NMBAs) is often restricted to enable intraoperative motor evoked potential monitoring [1]. Moreover, during cranial surgery, it is often difficult to attach sensors for processed electroencephalogram monitoring, which is used to monitor anesthetic depth [2]. Intraoperative coughing can lead to vigorous body movements and consequently movement-induced injury to surgically manipulated tissues, such as the brain and spinal cord in neurosurgical patients [3] Intraoperative coughing can also cause serious damage to the skull or scalp when a skull clamp is applied to fix the head position [4]. Intraoperative coughing can also increase the difficulty of cranial surgery by increasing the intracranial pressure and making the brain bulge [5].

Causes of intraoperative coughing under general anesthesia include too light anesthesia, inadequate local anesthesia in the larynx, insufficient neuromuscular blockade, movement of the endotracheal tube or patient’s head, inflation or deflation of the endotracheal cuff, and endotracheal or endobronchial suction [6]. However, no studies have analyzed clinical data to identify the incidence and predictors of intraoperative coughing specifically in neurosurgical patients.

The endotracheal cuff pressure is positively correlated with the airway pressure during positive-pressure ventilation [7]. Increased peak inspiratory pressure (PIP) is related to increased endotracheal cuff pressure during laparoscopic surgery and a high endotracheal cuff pressure increases the incidence of postoperative respiratory complications, such as cough, sore throat, and hoarseness [7, 8]. We hypothesized that increased endotracheal cuff pressure due to increased airway pressure might irritate the tracheal mucosa and evoke intraoperative coughing more frequently.

Therefore, this retrospective study investigated the incidence and timing of intraoperative coughing and evaluated the association between a high PIP and intraoperative coughing (primary outcome measure) in neurosurgical patients undergoing general anesthesia without neuromuscular blockade.

## Methods

### Ethics

Prior to data collection, the Institutional Review Board of Seoul National University College of Medicine/Seoul National University Hospital approved this study (number: H-2201–064–1290, date: January 24, 2022) and waived the requirement for informed consent. This paper adhered to the Strengthening the Reporting of Observational Studies in Epidemiology statement and all methods were carried out in accordance with the Declaration of Helsinki.

### Subjects

This retrospective study involved patients who underwent neurosurgery under total intravenous anesthesia (TIVA) with tracheal intubation and should not have received neuromuscular blockade after tracheal intubation between December 2017 and November 2019. Patients who had no data from the ventilator in the registry file, underwent relatively light anesthesia (intentionally maintaining a bispectral index [BIS] of around 60) for deep brain stimulation, or received additional NMBAs during surgery were excluded.

### Data collection

All data were collected retrospectively. Demographic (sex, age, height, and weight), preoperative (American Society of Anesthesiologists physical status and comorbidities including hypertension, diabetes mellitus, asthma, chronic obstructive pulmonary disease, smoking, obesity, cardiac disease, pulmonary disease, hepatic disease, and renal disease), surgical (type, position, and duration), and anesthetic (administration of NMBAs) data were obtained from electronic medical records. Continuous data from the ventilator (PIP, positive end-expiratory pressure [PEEP], and tidal volume [TV]), infusion pumps (effect site concentration [Ce] of propofol and remifentanil), and BIS monitor (BIS and frontal electromyogram [fEMG]) were obtained from a data registry (Vital Recorder ver. 1.8.15.5; Vital DB, Seoul, Korea) that stores these data automatically with a temporal resolution of 500 Hz [9]. In patients with intraoperative coughing, continuous data from the registry file were extracted for 1 min immediately before the onset of intraoperative coughing, whereas in patients without intraoperative coughing, they were extracted for 1 min randomly selected from the entire surgical duration to reduce selection bias and reflect the clinical situation at various time points during surgery. If intraoperative coughing was observed multiple times in the same patient, these data were extracted only for 1 min immediately before the first occurrence of intraoperative coughing. These data were averaged and used for analysis.

### Intraoperative coughing

Intraoperative coughing was usually documented in the anesthetic records. Intraoperative coughing was identified by checking the anesthetic records for a record of intraoperative coughing and comprehensively analyzing several waveforms (capnogram, plethysmogram, and the airway, arterial, and central venous pressure waveforms) recorded in the registry files (Additional file 1). The registry files of all patients were screened to find any intraoperative coughing that was missing from the anesthetic records. When screening intraoperative coughing in the registry file, we first checked whether there was a moment when PIP increased rapidly during surgery. If present, then we checked for irregular changes in several waveforms (e.g., a notch in the capnogram, a shake on the plethysmogram, an inspiratory airway pressure waveform below PEEP, and a shake on arterial or central venous pressure waveform, if present) at that point. When it was difficult to determine whether intraoperative coughing was present by analyzing the waveforms, another anesthesiologist was asked to make a decision by analyzing the waveforms. The time point at which PIP began to rise abruptly was regarded as the onset of intraoperative coughing.

If intraoperative coughing occurred, mechanical ventilation was stopped to avoid additional dyssynchrony with the ventilator and intravenous delivery of propofol and remifentanil was checked. After that, the target Ce of propofol and remifentanil was raised to suppress intraoperative coughing. Administration of NMBAs was not allowed because of motor evoked potential monitoring.

### Anesthetic management

Without premedication, patients were monitored with noninvasive blood pressure measurement, peripheral pulse oximetry, and electrocardiography in the operating room. TIVA was induced with a target-controlled infusion of remifentanil (Ce = 4 ng/mL) and propofol (Ce = 4 µg/mL) using the Minto and Schnider models, respectively. Rocuronium (0.6–0.8 mg/kg) was administered only once during anesthetic induction to facilitate tracheal intubation and was not administered thereafter, in order to monitor intraoperative motor evoked potentials. The upper airway including larynx was not topically anesthetized. A reinforced endotracheal tube with an inner diameter of 7.5–8.0 mm was inserted in males and 7.0 mm in females. The endotracheal cuff was inflated to a manometer pressure between 20 and 30 cmH_2_O. The endotracheal cuff pressure was not measured again or adjusted unless cuff leak was suspected. Mechanical ventilation was routinely maintained in volume-controlled mode with a TV of 8 mL/kg based on the predicted body weight (PBW) and a PEEP of 5 cmH_2_O [10–12]. Arterial catheterization was performed for invasive blood pressure measurement, if necessary. Respiratory rate was controlled to maintain partial pressure of arterial carbon dioxide at 30–35 mmHg in patients with increased intracranial pressure or patients who needed brain relaxation, and at 35–45 mmHg in other patients. Target-controlled infusion of propofol and remifentanil was used to maintain anesthesia and the Ce of propofol and remifentanil was adjusted to maintain the BIS, if monitored, between 40 and 60 and mean arterial pressure at 80–120% of the preoperative baseline, respectively. If mean arterial pressure was continuously below 80% even after titration of remifentanil, fluid or vasopressor, such as phenylephrine and ephedrine, was administered. After anesthetic induction, the patient was positioned for ease of surgery.

### Outcomes

The primary outcome measure was the association between high PIP and intraoperative coughing. The incidence, timing, and other perioperative predictors of intraoperative coughing were also investigated.

### Propensity score matching

To determine whether PIP is independently related to intraoperative coughing, patients were divided into high and low PIP groups based on the optimal cutoff value of PIP for intraoperative coughing, which is defined as a value maximizing the sum of sensitivity and specificity in receiver operating characteristic (ROC) analysis. Then, propensity score matching was performed in a 1:1 ratio to minimize biases resulting from imbalance in covariate distribution between the high and low PIP groups. All variables investigated in this study were used for propensity score matching except for PIP, BIS value, and fEMG. The variables included in propensity score matching were sex, age, body mass index, American Society of Anesthesiologists physical status, hypertension, diabetes mellitus, asthma, chronic obstructive pulmonary disease, smoking, obesity, cardiac disease, pulmonary disease, hepatic disease, renal disease, surgical type, surgical position, surgical duration, BIS monitoring, Ce of propofol and remifentanil, PEEP, and TV/PBW. Estimation and matching algorithms for propensity score matching were logistic regression and nearest neighbor, respectively, and caliper was 0.01.

### Statistical analysis

The normality of data distributions was evaluated using the Shapiro–Wilk test. Data for categorical variables are expressed as number of patients (proportion) and compared using the Pearson’s χ^2^ test or Fisher’s exact test according to the expected frequency of cells. Data for continuous variables are expressed as mean (standard deviation) or median (interquartile range) and compared using the Student’s t-test or Mann–Whitney U-test according to the normality of their distribution. To identify independent predictors of intraoperative coughing, four (prone position, surgical duration, a high PIP (PIP > 21.6 cmH_2_O), and TV/PBW) and three (surgical duration, a high PIP (PIP > 21.6 cmH_2_O), and TV/PBW) variables with P values < 0.05 in the univariable logistic regression analysis were entered into a multivariable logistic regression analysis before and after propensity score matching respectively. ROC analysis was used to evaluate the discriminative power of predictors, which was categorized into five grades based on the area under the curve (AUC): 0.5–0.6, fail; 0.6–0.7, poor; 0.7–0.8, fair; 0.8–0.9, good; 0.9–1.0, excellent. [13] The optimal cutoff value of predictors was set to a value maximizing the sum of sensitivity and specificity. A P value < 0.05 was considered statistically significant. All statistical analyses were conducted using a statistical software (IBM® SPSS® statistics 25; International Business Machines Corporation, Armonk, NY).

## Results

**Fig. 1:**
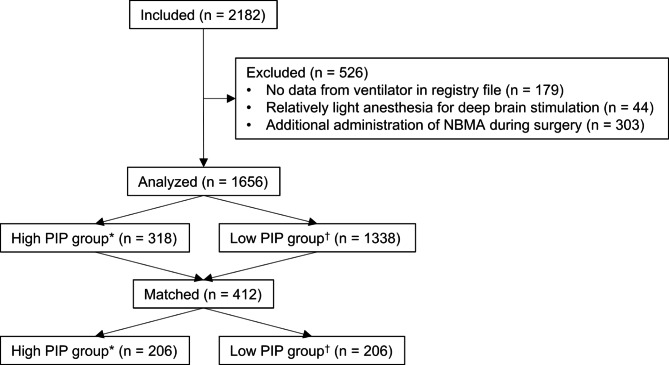
Study flow diagram *PIP > 21.6 cmH_2_O. ^†^PIP ≤ 21.6 cmH_2_O. DBS, deep brain stimulation; NMBA, neuromuscular blocking agent; PIP, peak inspiratory pressure

This study considered 2182 patients who underwent neurosurgery under TIVA with tracheal intubation (Fig. 1). Of these, 179, 44, and 303 patients were excluded from this study because of no data from the ventilator in the registry file, relatively light anesthesia for deep brain stimulation, and additional administration of NMBAs during surgery, respectively. Finally, 1656 patients were available for the data analysis. Among them, BIS and fEMG were monitored in 662 (40.0%) patients.

Intraoperative coughing was observed in 30 (1.8%) patients, once in 29 (1.8%) patients and twice in 1 (0.1%) patient. Intraoperative coughing occurred before, during, and after the main surgical procedure (from the end of dural opening to the beginning of dural closure in craniotomy, from the end of scalp opening to the end of skull flap fixation in cranioplasty, from the end of burr hole opening to the beginning of cranial wound closure in ventriculoperitoneal shunt, from the beginning of laminectomy to the beginning of wound closure in spinal tumor surgery, and from the beginning of laminectomy to the end of pedicle screw insertion in thoracolumbar interbody fusion) in 14 (0.8%), 9 (0.5%), and 7 (0.4%) patients, respectively. There were nine patients who needed additional assessment of the registry file by another anesthesiologist to confirm intraoperative coughing, and intraoperative coughing was finally identified in four patients of them.

In the ROC analysis of intraoperative coughing, PIP had an AUC of 0.66 (95% confidence interval [CI] 0.56–0.76, P = 0.003) and its optimal cutoff value was 21.6 cmH_2_O. Patients were divided into high (PIP > 21.6 cmH_2_O, n = 318) and low (PIP ≤ 21.6 cmH_2_O, n = 1338) PIP groups. After propensity score matching, all included variables matched well (P = 0.929) and no variables differed significantly between the high and low PIP groups (both n = 206), except for higher fEMG (29.1 [26.8–33.0] vs. 27.1 [26.2–30.6], P = 0.007) in the high PIP group (n = 80) than the low PIP group (n = 83, Table 1). The incidence of intraoperative coughing was significantly higher in the high PIP group than in the low PIP group before (14/318 [4.4%] vs. 16/1338 [1.2%], P < 0.001) and after (13/206 [6.3%] vs. 1/206 [0.5%], P = 0.003) propensity score matching.

**Table 1:** Comparisons of demographic, preoperative, and intraoperative data between the low and high PIP groups before and after propensity score matching

	Before propensity score matching			After propensity score matching		
	Low PIP group* (n = 1338)	High PIP group^†^ (n = 318)	P value	Low PIP group* (n = 206)	High PIP group^†^ (n = 206)	P value
Demographics						
Male sex	604 (45.1%)	150 (47.2%)	0.555	95 (46.1%)	93 (45.1%)	0.486
Age (year)	58.6 (46.8–67.2)	59.1 (47.4–67.8)	0.599	59.5 (48.6–68.0)	59.0 (46.5–68.9)	0.515
BMI (kg/m^2^)	24.0 (21.8–26.2)	26.3 (24.0-28.7)	< 0.001	25.1 (23.0-27.8)	25.5 (23.5–28.2)	0.390
ASA physical status			0.004			0.382
1	297 (22.2%)	42 (13.2%)	< 0.001	36 (17.5%)	32 (15.5%)	0.691
2	936 (70.0%)	246 (77.4%)	0.011	159 (77.2%)	157 (76.2%)	0.907
3	92 (6.9%)	29 (9.1%)	0.207	10 (4.9%)	17 (8.3%)	0.232
4	12 (0.9%)	1 (0.3%)	0.483	1 (0.5%)	0 (0.0%)	1.000
5	1 (0.1%)	0 (0.1%)	1.000	0 (0.0%)	0 (0.0%)	NA
Comorbidity						
Hypertension	420 (31.4%)	118 (37.1%)	0.059	68 (33.0%)	73 (35.4%)	0.678
Diabetes mellitus	177 (13.2%)	57 (17.9%)	0.038	34 (16.5%)	31 (15.0%)	0.787
Asthma	17 (1.3%)	6 (1.9%)	0.422	5 (2.4%)	4 (1.9%)	1.000
COPD	15 (1.1%)	5 (1.6%)	0.565	2 (1.0%)	3 (1.5%)	1.000
Smoking	213 (15.9%)	78 (24.5%)	< 0.001	37 (18.0%)	40 (19.4%)	0.800
Obesity	60 (4.5%)	54 (17.0%)	< 0.001	28 (13.6%)	18 (8.7%)	0.159
Cardiac disease	90 (6.7%)	33 (10.4%)	0.035	18 (8.7%)	21 (10.2%)	0.736
Pulmonary disease	69 (5.2%)	19 (6.0%)	0.656	11 (5.3%)	12 (5.8%)	1.000
Hepatic disease	73 (5.5%)	27 (8.8%)	0.035	19 (9.2%)	16 (7.8%)	0.724
Renal disease	64 (4.8%)	18 (5.7%)	0.614	8 (3.9%)	11 (5.3%)	0.639
Surgery						
Type			0.002			0.420
Cranial	950 (71.0%)	197 (61.9%)		120 (58.3%)	129 (62.6%)	
Spinal	388 (29.0%)	121 (38.1%)		86 (41.7%)	77 (37.4%)	
Position			0.117			0.109
Supine	906 (67.7%)	196 (61.6%)	0.046	123 (50.0%)	123 (50.0%)	1.000
Prone	398 (29.7%)	113 (35.5%)	0.052	79 (38.3%)	71 (34.5%)	0.474
Lateral	34 (2.5%)	9 (2.8%)	0.924	4 (1.9%)	12 (5.8%)	0.074
Duration (min)	165.0 (105.0-250.0)	185.5 (115.0-250.0)	0.015	170.0 (105.0-251.3)	183.0 (105.0-260.0)	0.622
Duration > 200.5 min	495 (37.0%)	143 (45.0%)	0.009	78 (37.9%)	88 (42.7%)	0.315
Anesthesia						
BIS monitoring	545 (40.7%)	117 (36.8%)	0.220	83 (40.3%)	80 (38.8%)	0.840
BIS value	38.4 (29.9–45.3)	38.7 (28.4–45.9)	0.800	38.9 (31.0-43.4)	38.4 (28.8–45.5)	0.963
fEMG (dB)	27.9 (26.5–30.9)	28.7 (26.6–32.7)	0.083	27.1 (26.2–30.6)	29.1 (26.8–33.0)	0.007
Ce						
Propofol (µg/kg)	3.5 (3.0–4.0)	3.5 (2.8-4.0)	0.363	3.5 (2.5-4.0)	3.5 (2.7–4.3)	0.173
Remifentanil (ng/kg)	3.5 (2.8-4.0)	3.5 (2.8–4.1)	0.623	3.5 (2.6-4.0)	3.5 (3.0-4.2)	0.367
Ventilation						
PIP (cmH_2_O)	18.0 (16.0-19.4)	23.1 (22.1–24.9)	< 0.001	19.0 (17.2–20.1)	23.0 (22.0-24.1)	< 0.001
PEEP (cmH_2_O)	5.0 (5.0–5.0)	7.1 (5.0-8.1)	< 0.001	5.1 (5.0-7.9)	5.1 (5.0-7.9)	0.660
TV/PBW (mL/kg)	7.5 (7.0-7.8)	7.7 (7.2–8.1)	< 0.001	7.6 (7.2-8.0)	7.6 (7.2-8.0)	0.889
TV/PBW > 7.9 mL/kg	276 (20.6%)	108 (34.0%)	< 0.001	55 (26.7%)	64 (31.1%)	0.328
Intraoperative coughing	16 (1.2%)	14 (4.4%)	< 0.001	1 (0.5%)	13 (6.3%)	0.003

Data are expressed as number of patients (proportion) or median (interquartile range). *PIP ≤ 21.6 cmH_2_O. ^†^PIP > 21.6 cmH_2_O. ASA, American Society of Anesthesiologists; COPD, chronic obstructive pulmonary disease; PIP, peak inspiratory pressure; BMI, body mass index; BIS, bispectral index; fEMG, frontal electromyogram; Ce, effect site concentration; PEEP, positive end-expiratory pressure; TV/PBW, tidal volume divided by predicted body weight; NA, not applicable

After propensity score matching, PIP (22.9 [22.0–24.3] vs. 21.4 [19.0–23.0] cmH_2_O, P = 0.007) and TV/PBW (8.0 [7.4–9.0] vs. 7.6 [7.2–8.7] mL/kg, P = 0.047) were significantly higher and surgical duration (231.5 [193.3–320.0] vs. 170.5 [105.0-255.0] min, P = 0.015) was significantly longer in patients with intraoperative coughing than in those without (Table 2). In a multivariable logistic regression analysis, a high PIP (odds ratio [95% CI] 14.22 [1.81–111.73], P = 0.012), TV/PBW (mL/kg, 1.36 [1.09–1.69], P = 0.006), and surgical duration (min, 1.01 [1.00–1.01], P = 0.025) were significantly associated with intraoperative coughing (Table 3). In the ROC analysis of intraoperative coughing, the AUC of TV/PBW and surgical duration were 0.66 (95% CI 0.51–0.80, P = 0.047) and 0.69 (95% CI 0.57–0.81) and their optimal cutoff values were 7.9 mL/kg and 200.5 min, respectively (Table 4).

**Table 2:** Comparisons of demographic, preoperative, and intraoperative data between patients with and without intraoperative coughing before and after propensity score matching

	Before propensity score matching			After propensity score matching		
	Patients with intraoperative coughing (n = 30)	Patients without intraoperative coughing (n = 1626)	P value	Patients with intraoperative coughing (n = 14)	Patients without intraoperative coughing (n = 398)	P value
Demographics						
Male sex	14 (46.7%)	740 (45.5%)	1.000	7 (50.0%)	171 (43.0%)	0.804
Age (year)	61.6 (48.2–67.9)	58.7 (46.9–67.5)	0.412	64.6 (56.9–68.6)	59.0 (47.3–68.6)	0.179
BMI (kg/m^2^)	25.8 (21.4–27.6)	24.4 (22.1–26.7)	0.885	25.8 (21.8–28.1)	25.3 (23.4–28.1)	0.603
ASA physical status			0.208			0.993
1	3 (10.0%)	336 (20.7)	0.228	2 (14.3%)	66 (16.6%)	1.000
2	25 (83.3%)	1157 (71.2%)	0.144	11 (78.6%)	305 (76.6%)	1.000
3	2 (6.7%)	119 (7.3%)	1.000	1 (7.1%)	26(6.5%)	1.000
4	0 (0.0%)	13 (0.8%)	1.000	0 (0.0%)	1 (0.3%)	1.000
5	0 (0.0%)	1 (0.1%)	1.000	0 (0.0%)	0 (0.0%)	NA
Comorbidity						
Hypertension	12 (40.0%)	526 (32.3%)	0.490	6 (42.9%)	135 (33.9%)	0.569
Diabetes mellitus	8 (26.7%)	226 (13.9%)	0.060	4 (28.6%)	61 (15.3%)	0.251
Asthma	0 (0.0%)	23 (1.4%)	1.000	0 (0.0%)	9 (2.3%)	1.000
COPD	1 (3.3%)	19 (1.2%)	0.308	0 (0.0%)	5 (1.3%)	1.000
Smoking	2 (6.7%)	289 (17.8%)	0.180	1 (7.1%)	76 (19.1%)	0.483
Obesity	0 (0.0%)	114 (7.0%)	0.262	0 (0.0%)	46 (11.6%)	0.383
Cardiac disease	5 (16.7%)	118 (7.3%)	0.066	3 (21.4%)	36 (9.0%)	0.137
Pulmonary disease	3 (10.0%)	85 (5.2%)	0.211	0 (0.0%)	23 (5.8%)	1.000
Hepatic disease	4 (13.3%)	97 (6.0%)	0.106	1 (7.1%)	34 (8.5%)	1.000
Renal disease	4 (13.3%)	78 (4.8%)	0.057	2 (14.3%)	17 (4.3%)	0.132
Surgery						
Type			0.235			0.257
Cranial	24 (80.0%)	1123 (69.1%)		11 (78.6%)	238 (59.8%)	
Spinal	6 (20.0%)	503 (30.9%)		3 (1.8%)	160 (40.2%)	
Position			0.054			0.201
Supine	24 (80.0%)	1078 (66.3%)	0.167	11 (78.6%)	235 (59.0%)	0.253
Prone	4 (13.3%)	507 (31.2%)	0.058	2 (14.3%)	148 (37.2%)	0.142
Lateral	2 (6.7%)	41 (2.5%)	0.404	1 (7.1%)	15 (3.8%)	0.431
Duration (min)	220.0 (170.0-299.0)	168.0 (106.8–255.0)	0.006	231.5 (193.3–320.0)	170.5 (105.0-255.0)	0.015
Duration > 200.5 min	619 (38.1%)	19 (63.3%)	0.005	155 (38.9%)	11 (78.6%)	0.003
Anesthesia						
BIS monitoring	11 (36.7%)	651 (40.0%)	0.851	2 (14.3%)	161 (40.5%)	0.091
BIS value	46.3 (34.7–59.1)	38.4 (29.8–45.2)	0.069	45.6 (45.5, 45.6)*	38.5 (28.6–43.5)	NA
fEMG (dB)	34.3 (28.1–38.2)	27.9 (26.5–31.2)	0.007	47.5 (45.9, 49.0)*	27.8 (26.4–32.1)	NA
Ce						
Propofol (µg/kg)	3.5 (3.0-4.2)	3.5 (3.0–4.0)	0.747	4.0 (2.9–4.6)	3.5 (2.5-4.0)	0.139
Remifentanil (ng/kg)	3.9 (3.0-4.6)	3.5 (2.8-4.0)	0.242	3.9 (3.0–6.0)	3.5 (2.8-5.0)	0.184
Ventilation						
PIP (cmH_2_O)	20.6 (18.0-23.2)	18.4 (16.8–20.9)	0.003	22.9 (22.0-24.3)	21.4 (19.0–23.0)	0.007
PIP > 21.6 cmH_2_O	14 (46.7%)	304 (18.7%)	< 0.001	13 (92.9%)	193 (48.5%)	0.003
PEEP (cmH_2_O)	5.1 (5.0-5.6)	5.0 (5.0–6.0)	0.553	5.4 (4.9–8.2)	5.1 (5.0–8.0)	0.719
TV/PBW (mL/kg)	7.8 (7.3–8.6)	7.5 (7.0-7.9)	0.006	8.0 (7.4-9.0)	7.6 (7.2–8.7)	0.047
TV/PBW > 7.9 mL/kg	14 (46.7%)	370 (22.8%)	0.004	8 (57.1%)	111 (27.9%)	0.031

Data are expressed as number of patients (proportion) or median (interquartile range). *: median (range). BMI, body mass index; ASA, American Society of Anesthesiologists; COPD, chronic obstructive pulmonary disease; BIS, bispectral index; fEMG, frontal electromyogram; Ce, effect site concentration; PIP, peak inspiratory pressure; PEEP, positive end-expiratory pressure; TV/PBW, tidal volume divided by predicted body weight; NA, not applicable because of little data in patients with intraoperative coughing

**Table 3:** Univarable and multivariable logistic regression analyses for intraoperative coughing

	Before propensity score matching				After propensity score matching			
	Univariable logistic regression analysis		Multivariable logistic regression analysis*		Univariable logistic regression analysis		Multivariable logistic regression analysis^†^	
	OR (95% CI)	P value	OR (95% CI)	P value	OR (95% CI)	P value	OR (95% CI)	P value
Prone position	0.34 (0.12–0.98)	0.045	0.35 (0.12–1.04)	0.058				
Surgical duration (min)	1.00 (1.00-1.01)	0.033	1.00 (1.00-1.01)	0.192	1.00 (1.00-1.01)	0.047	1.01 (1.0-1.01)	0.025
PIP > 21.6 cmH_2_O	3.81 (1.83–7.88)	< 0.001	3.21 (1.50–6.88)	0.003	13.81 (1.79-106.56)	0.012	14.22 (1.81-111.73)	0.012
TV/PBW (mL/kg)	1.45 (1.21–1.74)	< 0.001	1.36 (1.11–1.66)	0.003	1.34 (1.10–1.64)	0.004	1.36 (1.09–1.69)	0.006

*The adjusted variables were prone position, surgical duration, PIP > 21.6 cmH_2_O, and TV/PBW. Nagelkerke R^2^ and P value of Hosmer-Lemeshow goodness of fit test were 0.096 and 0.811, respectively. ^†^The adjusted variables were surgical duration, PIP > 21.6 cmH_2_O, and TV/PBW. Nagelkerke R^2^ and P value of Hosmer-Lemeshow goodness of fit test were 0.209 and 0.950, respectively. OR, odds ratio; CI, confidence interval; PIP, peak inspiratory pressure; TV/PBW, tidal volume divided by predicted body weight

**Table 4:** Receiver operating characteristic analysis

	Before propensity score matching					After propensity score matching				
	AUC (95% CI)	P value	Optimal cutoff value	Sensitivity	Specificity	AUC (95% CI)	P value	Optimal cutoff value	Sensitivity	Specificity
PIP (cmH_2_O)	0.66 (0.56–0.76)	0.003	21.6	46.7%	81.3%	0.71 (0.60–0.83)	0.007	21.6	92.9%	51.5%
TV/PBW (mL/kg)	0.65 (0.54–0.75)	0.006	7.9	46.7%	77.2%	0.66 (0.51–0.80)	0.047	7.9	57.1%	72.1%
Surgical duration (min)	0.65 (0.56–0.73)	0.006	169.5	80.0%	50.2%	0.69 (0.57–0.81)	0.015	200.5	78.6%	61.1%

AUC, area under the curve; CI, confidence interval; PIP, peak inspiratory pressure; TV/PBW, tidal volume divided by predicted body weight

## Discussion

With the increasing use of intraoperative neurophysiological monitoring, including motor evoked potential monitoring, in neurosurgery, the use of NMBAs is frequently restricted during surgery. Therefore, intraoperative coughing is prone to occur during neurosurgery and it is a major concern for anesthesiologists and neurosurgeons. This retrospective study examined the incidence and predictors of intraoperative coughing in neurosurgical patients who underwent general anesthesia without neuromuscular blockade and found intraoperative coughing in 1.8% of patients. A high PIP was a significant predictor of intraoperative coughing in these patients.

In clinical practice, intraoperative coughing should be avoided in neurosurgical patients, because it can lead to patient movement, resulting in serious damage to the brain and spinal cord. [3] Unfortunately, there has been no literature definitely describing the incidence and risk factors of intraoperative coughing in neurosurgical patients. Although few previous studies have reported intraoperative coughing with an incidence of 0.0–0.9% during neurosurgery under total intravenous anesthesia without additional neuromuscular blockade after tracheal intubation, only intraoperative coughing caused by stimuli for motor evoked potential monitoring was considered in these previous studies [14, 15]. Unlike the aforementioned studies, this study evaluated the occurrence of intraoperative coughing throughout the neurosurgery and showed its incidence of 1.8% during the entire surgical duration and 0.5% during the main surgical procedure.

In this study, patients with PIP > 21.6 cmH_2_O had a 14.2-fold higher risk for intraoperative coughing than those with PIP ≤ 21.6 cmH_2_O. Although there has been no previous report to provide a direct evidence of the relationship between PIP and intraoperative coughing, this relationship may be partially explained by the endotracheal cuff pressure, which may be elevated by a high PIP. In two gynecological laparoscopic surgery studies, the endotracheal cuff pressure changed significantly with the airway pressure during peritoneal insufflation and deflation and a high endotracheal cuff pressure was associated with postoperative respiratory complications [7, 16]. It is necessary to maintain a relatively high endotracheal cuff pressure to avoid air leakage during positive-pressure ventilation in patients with a high PIP [17]. Therefore, patients with a high PIP likely had relatively high endotracheal cuff pressures, resulting in greater irritation to the tracheal mucosa and more frequent intraoperative coughing. On the other hand, smoking, asthma, and chronic obstructive pulmonary disease, which are generally thought to increase airway resistance and cause coughing, were not significantly related to intraoperative coughing both before and after propensity score matching.

A high TV/PBW was also associated with intraoperative coughing in this study. This association was significant even after adjusting for PIP in the multivariable logistic regression analysis. A high TV accompanies a high flow on the upper airway, which can lead to coughing by increasing airway irritation. Also, a high TV can cause hyperinflation of the lungs, which can provoke the Hering–Breuer reflex, in which excessive lung inflation evokes reflexive expiration to prevent lung injury from volutrauma [18]. Therefore, we speculate that patients with a high TV/PBW may have an increased chance of airway irritation and reflexive expiration, which can be seen as coughing during mechanical ventilation.

A long surgical duration was also related to intraoperative coughing in this study. The relationship between intraoperative coughing and surgical duration can be explained easily by the cumulative risk for intraoperative coughing. There is also a possibility of increased microaspiration, which may evoke coughing, as surgical duration increases [19].

The prone position was related to a lower risk of intraoperative coughing only in univariable logistic regression analysis before propensity score matching. There are two possibilities to explain this finding. First, the patient’s body and head positions affect the endotracheal cuff pressure. Although studies have inconsistent results regarding whether the endotracheal cuff pressure increases in the prone position compared to the supine position, heads in the flexed, extended, and rotated positions other than the neutral position increase the endotracheal cuff pressure [20–24]. In our experience, a near-neutral head position is more common in surgery performed in the prone position (e.g., posterior thoracolumbar spinal surgery), than in surgery performed in the supine position (e.g., cranial surgery). Differences in the endotracheal cuff pressure according to the patient’s head position, which was not investigated in this study, may have partly contributed to the difference in the incidence of intraoperative coughing. Second, in the prone position, the possibility of microaspiration may be lower than in other positions, particularly the supine position, because secretions flow out of the oral cavity rather than pooling in the oral cavity [19, 25, 26].

In patients with BIS monitoring, the fEMG was higher in patients with intraoperative coughing than in those without. fEMG reflects both the activity of the frontalis muscle and subcortical activity, which indicates nociception during anesthesia. In a previous study, the difference between response entropy and state entropy, reflecting fEMG activity, increased with the intensity of electrical stimulation [27]. This difference was successfully used as an indicator to guide remifentanil administration and avoid unwanted responses in other previous studies [28, 29]. Thus, the high fEMG before intraoperative coughing in this study suggests that intense nociception from surgical stimulation under insufficient analgesia may contribute to intraoperative coughing. In the same context, a previous study introduced facial nerve electromyogram, rather than BIS, as an effective monitor for predicting patient movement during craniofacial and skull base surgeries [30].

This study had several limitations. First, potential biases, such as selection and information bias, existed because of the retrospective study design. Second, the explanatory power of multivariable logistic regression analysis was relatively weak. It is possible that other significant predictors of intraoperative coughing were missing in this study. Additional information on preoperative (prescription drugs, neurologic deficit, and surgical indication) and intraoperative (endotracheal cuff pressure, head position, intensity of surgical stimulation, partial pressure of end-tidal carbon dioxide, peripheral oxygen saturation, mean arterial pressure, heart rate, body temperature, and vasopressor administration) variables can help elucidate the exact mechanism of intraoperative coughing. Third, because the patients in this study underwent TIVA without additional administration of NMBAs during surgery, it is difficult to apply our results to neurosurgical patients who received NMBAs during surgery or patients who undergo inhalational anesthesia. Fourth, because the endotracheal cuff pressure was not measured continuously or strictly controlled throughout the surgery in this study, whether a high PIP results in intraoperative coughing by increased endotracheal cuff pressure is not fully investigated. Fifth, PIP and other variables were extracted for only one minute immediately before intraoperative coughing. This PIP may not be representative of the rest of the intraoperative period before coughing. Also, we compared the PIP for one minute immediately before intraoperative coughing with the PIP at a random point during the surgery in patients without intraoperative coughing. Such a comparison can be biased. We did not know the optimal time period and frame to predict intraoperative coughing in this study. The data extraction from a long time period and from a time frame further away from intraoperative coughing can help predict intraoperative coughing. Lastly, because rocuronium is an intermediate-acting NMBA, the effect of rocuronium administered only for tracheal intubation may have remained to some extent after the start of surgery if the time from tracheal intubation to the start of surgery is short. Therefore, there was a possibility that the residual effect of rocuronium suppressed intraoperative coughing in the early intraoperative period.

## Conclusions

The incidence of intraoperative coughing was 1.8% in neurosurgical patients under general anesthesia without neuromuscular blockade. A high PIP may be associated with intraoperative coughing in such patients.

## Electronic supplementary material

Below is the link to the electronic supplementary material.

Supplementary Material 1

## Data Availability

The datasets used and/or analyzed during the current study are available from the corresponding author on reasonable request.

## Abbreviations

ASA: American Society of Anesthesiologists
AUC: area under the curve
BIS: bispectral index
BMI: body mass index
Ce: effect site concentration
CI: confidence interval
COPD: chronic obstructive pulmonary disease
fEMG: frontal electromyogram
NA: not applicable
NMBA: neuromuscular blocking agent
OR: odds ratio
PBW: predicted body weight
PEEP: positive end-expiratory pressure
PIP: peak inspiratory pressure
ROC: receiver operating characteristic
TIVA: total intravenous anesthesia
TV: tidal volume
TV/PBW: tidal volume divided by predicted body weight
