# Supplementary material for: High peak inspiratory pressure may be associated with intraoperative coughing during neurosurgery under general anesthesia without neuromuscular blockade: a retrospective study
Source: BMC Anesthesiol. 2023 Apr 14;23:123. doi: 10.1186/s12871-023-02080-6 (PMC10103441; doi:10.1186/s12871-023-02080-6)

Additional file 1. Identification of intraoperative coughing in a registry file

A: sudden increases in airway pressures. B: decreases in airway pressures below positive end-expiratory pressure. C: notches in capnogram. D: shakes on arterial pressure waveform. E: shakes on plethysmogram.


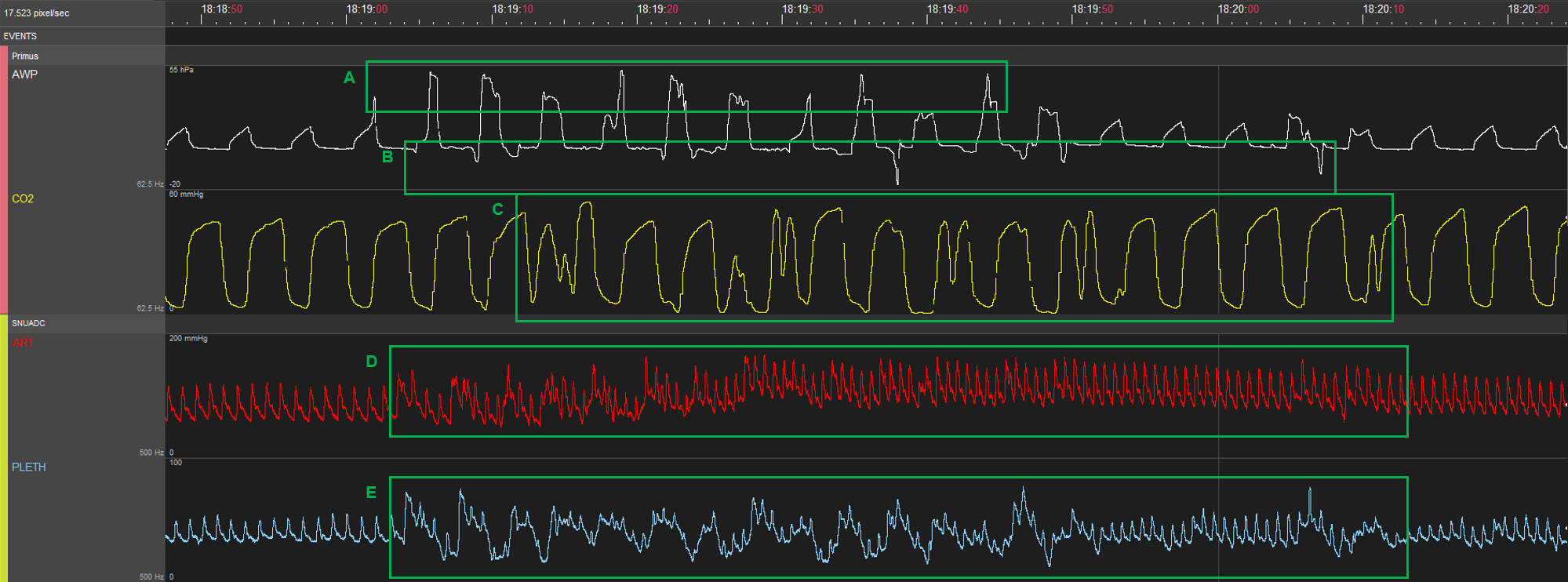

Supplement: Supplementary file 1 — Supplementary Material 1 [file 12871_2023_2080_MOESM1_ESM.docx]
